# *TP53* signature diagnostic system using multiplex reverse transcription–polymerase chain reaction system enables prediction of prognosis of breast cancer patients

**DOI:** 10.1007/s12282-021-01250-z

**Published:** 2021-07-24

**Authors:** Shin Takahashi, Takafumi Fukui, Tadashi Nomizu, Yoichiro Kakugawa, Fumisyoshi Fujishima, Takanori Ishida, Noriaki Ohuchi, Chikashi Ishioka

**Affiliations:** 1 Department of Medical Oncology, Tohoku University Hospital, 1-1, Seiryomachi, Aoba-ku, 4-1 Seiryo-machi, Aobaku, Sendai, 980-8575 Japan; 2 Department of Clinical Oncology, Tohoku University Graduate School of Medicine, 4-1, Seiryomachi, Aoba-ku, Sendai, Japan; 3 Biomedical Business Division, FALCO Biosystems Ltd., 346, Shimizu-cho Nijoagaru Kawaramachi-dori, Nakagyo-ku, Kyoto, Japan; 4 Department of Surgery, Hoshi General Hospital, 159-1, Mukaigawaramachi, Koriyama, Fukushima Japan; 5 Department of Breast Oncology, Miyagi Cancer Center Hospital, 47-1, Nodayama, Shiote, Medeshima, Natori, Japan; 6 Department of Surgery, Japanese Red Cross Sendai Hospital, 2-43-3, Yagiyama hon-cho, Taihaku-ku, Sendai, Miyagi Japan; 7 Department of Pathology, Tohoku University Hospital, 1-1, Seiryomachi, Aoba-ku, Sendai, Japan; 8 Department of Breast and Endocrine Surgical Oncology, Tohoku University Graduate School of Medicine, 1-1, Seiryomachi, Aoba-ku, Sendai, Japan

**Keywords:** *TP53* signature, Breast cancer, Prognostic factor, Diagnostic system

## Abstract

**Background:**

*TP53* status based on *TP53* signature, a gene expression profile to determine the presence or absence of *TP53* mutation, is an independent prognostic factor of breast cancer. The purpose of this study was to develop a simple diagnostic system for *TP53* signature status.

**Methods:**

We developed a multiplex reverse transcription–polymerase chain reaction system to determine *TP53* status. Based on this system, prospectively collected 189 patients with stage I and II breast cancer were determined to have *TP53* mutant signature or *TP53* wild-type signature. The prognostic significance of the *TP53* signature by the diagnostic system was analyzed.

**Results:**

The diagnostic accuracy of *TP53* status and reproducibility of this diagnosis system was confirmed. Using the diagnostic system, 89 patients were classified as *TP53* mutant signature and the remaining 100 cases were classified as *TP53* wild-type signature. Recurrence-free survival (RFS) among patients with *TP53* mutant signature was significantly shorter than that among those with *TP53* wild-type signature. On univariate and multivariate analyses, the *TP53* signature status was an independent predictor of RFS. RFS among patients with *TP53* mutant signature was significantly shorter than that among those with *TP53* wild-type signature in a cohort of estrogen receptor-positive breast cancer. Although a difference was not significant, no recurrent cases was observed in *TP53* wild-type signature group in triple negative breast cancer.

**Conclusion:**

This simple and precise diagnostic system to determine *TP53* signature status may help in prognostic assessment, therapeutic decision-making, and treatment optimization in patients with breast cancer.

**Supplementary Information:**

The online version contains supplementary material available at 10.1007/s12282-021-01250-z.

## Introduction

The plethora of comprehensive gene expression analyses in the context of breast cancer has gradually helped unravel the molecular biology of breast cancer. In addition, a large number of gene expression profiles that predict prognosis, recurrence, and therapeutic response to anticancer drugs and endocrine therapies has been reported [1]. Representative gene expression profiles, such as Onocotype DX [2–4], Mammaprint [5, 6], and Prosigna [7, 8], have already been approved by US Food and Drug Administration.

Tumor suppressor gene *TP53* is the most frequently mutated gene in human cancers, and the patients with *TP53* mutations are known to have poor clinical outcomes [9]. Several large-scale meta-analyses have shown *TP53* mutation to be an independent predictor of poor prognosis for breast cancer [10, 11]. Furthermore, *TP53* status is a predictive factor for chemotherapy [12, 13].

We had earlier found a gene expression signature (*TP53* signature) that correlates with presence or absence of *TP53* mutation [14]. The *TP53* status determined using the *TP53* signature was a prognostic factor independent of other known clinicopathological prognostic factors. Also, the *TP53* status determined using gene expression signature was a superior predictor of prognosis compared with that determined using immunohistochemical examination and direct DNA sequencing. Similar results were earlier reported by Miller et al. [15].

The purpose of this study was to develop a simple diagnostic system for *TP53* signature using multiplex reverse transcription–polymerase chain reaction (RT–PCR), to test its diagnostic precision and prognostic predictability in a prospective cohort and to examine the clinical significance of *TP53* signature among breast cancer subtypes.

## Patients and methods

### Patients and tumor tissues

This study was approved by the Ethics Committee at the Tohoku University Hospital (TU), Hoshi General Hospital (HG), and Miyagi Cancer Center (MCC). The TU cohort, which was used in our previous study [14], was used for the development of the *TP53* signature diagnosis system. Validation cohort is a breast cancer case series from HG and MCC prospectively from September, 2007 to October, 2013 [16]. None of the cases received chemotherapy or endocrine therapy preoperatively. Written informed consent for the study was obtained from all patients. A part of the surgical specimen of breast cancer was stored as fresh frozen (FF) tissue and/or formalin-fixed paraffin embedded (FFPE) tissue. Among patients enrolled in this study, we selected curatively resected patients with stage I–II breast cancer. Patients with ductal carcinoma in situ, those with unknown histology or those with squamous cell carcinoma were excluded from the analysis. The validation cohort was used to assess the prognostic ability of the *TP53* signature diagnosis system.

### Clinicopathological characteristics

Clinicopathological characteristics data (pathological tumor size, pathological lymph node status, pathological stage, ER, PgR, HER2, Grade, Ki-67, adjuvant chemotherapy and adjuvant endocrine therapy) were obtained from medical records. For cases for which Ki-67 data were not available, immunohistological staining for Ki-67 was performed at the Department of Pathology, Tohoku University Hospital, using the MIB-1 antibody (Dako, Carpinteria, CA, USA).

### RNA extraction

The glass slide specimen with 10-µm thick sections of FF and FFPE tissue blocks were prepared. In reference to the HE stained specimen, tumor cells were collected from FF tissue or FFPE tissue by macrodissection technique. Total RNA was extracted from FF tissue or FFPE tissue with use of RNeasy mini kit (Qiagen, Valencia, CA, USA) or RNeasy FFPE kit (Qiagen, Valencia, CA, USA), respectively.

### TP53 signature diagnosis system

Genome Lab GeXP Genetic Analysis System (Beckman Coulter, Brea, CA) was used to obtain gene expression profile. To obtain the *TP53* signature gene set for GeXP, genes for which the average signal value in the raw data exceeded 1000 in the previous microarray data of the TU cohort [14] and which had less homolog genes were selected. Based on these criteria, 23 genes were chosen among *TP53* signature genes. Three genes were added to this gene set as internal control; as a result, a *TP53* signature gene set that comprised of 26 genes was established (Supplemental Table 1). Primers for reverse transcription (RT) and for PCR were designed using Genome Lab eXpress Designer GeXP Software (Beckman Coulter, Brea, CA). The multiplex reaction was optimized as per the manual and optimal primer concentrations determined. RT and PCR were performed with GenomeLab GeXP Start Kit (Beckman Coulter, Brea, CA) in accordance to the manual. The quantity of input RNA was 1 μg for FFPE tissues and 50 ng for the FF tissues.

### TP53 signature score

*TP53* status was determined by *TP53* signature score, which is the ratio of the sum of expression levels of 16 genes that were upregulated in tumors with *TP53* mutation to the sum of expression values of 7 genes downregulated in tumors with*TP53* mutation. The cutoff level for *TP53* signature score was determined by Receiver Operating Characteristic curve (ROC) analysis based on the *TP53* signature status by microarray of TU cohort [14]. When *TP53* signature score of a certain sample was greater than 1.11, the sample was labeled as *TP53* mutant signature.

### Outcomes

The primary end point of the study was recurrence-free survival (RFS), which was defined as the period from the date of surgery for breast cancer to the date on which tumor recurrence. Overall survival (OS) was defined as the period from the date of surgery for breast cancer to the date of death. Breast cancer-specific survival (BCSS) was defined as the period from the date of surgery for breast cancer to the date of death by breast cancer.

### Statistical analysis

All statistical analyses were performed using JMP Pro 14.3.0 (SAS Institute Japan Co., Ltd., Tokyo, Japan). Baseline characteristics of patients (except age) were assessed by chi-squared test. Kruskal–Wallis test was used for statistical analysis of age. Survival curves were made with Kaplan–Mayer method, and between-group differences assessed with log-rank test. Univariate and multivariate analyses (Cox proportional hazard model) were conducted to assess the association between clinicopathological factors and the *TP53* status for RFS. P value under 0.05 was considered indicative of a statistically significant difference. This study is registered in UMIN-CTR (http://www.umin.ac.jp/ctr/) (000005172).

## Results

### Patients for analysis

**Fig. 1:**
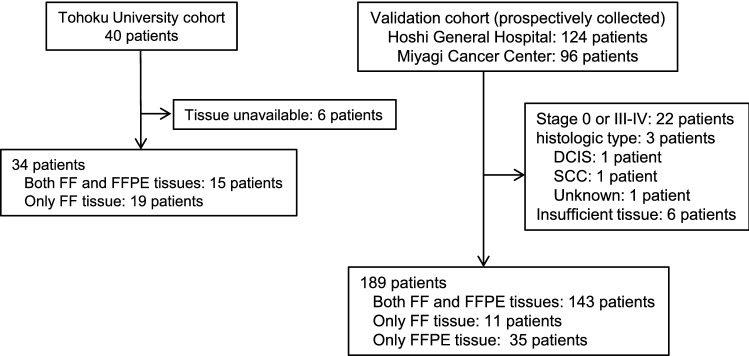
The details of the cohorts. The Tohoku University cohort comprises 40 patients, 34 of whom were included in the analysis. The validation cohort comprised 220 patients who underwent surgery. Out of the 220 patients, 31 were excluded based on the exclusion criteria (stage and histological type) or due to inadequate specimens. The remaining189 patients were included in the analysis. *FF* fresh frozen, *FFPE* formalin-fixed paraffin-embedded

The TU cohort comprises 40 patients, 34 of whom were included in this analysis. The validation cohort comprised 220 patients who had undergone surgery between October, 2013 and September, 2007. Out of the 220 patients, 31 patients were excluded based on the exclusion criteria (Fig. 1). The remaining 189 patients were included in the analysis. Median duration of observation period was 8.06 years (range 0.91–10.18 years).

### Cutoff value of TP53 signature score

RNAs extracted from 34 samples of the TU cohort were available for analysis. *TP53* signature of these 34 patients was examined with multiplex PCR method. From the result of ROC analysis, the cutoff value for *TP53* signature score was set at 1.11 (Area under the curve: AUC = 0.993) (Table 1).

**Table 1:** *TP53* status diagnosed by *TP53* signature score, microarray and Sanger sequence in the Tohoku University cohort

Sample	*TP53* signature score	*TP53* status by *TP53* signature score	*TP53* status by microarray
BR047	0.1707	Wild	Wild
BR038	0.3383	Wild	Wild
BR019	0.4267	Wild	Wild
BR044	0.5178	Wild	Wild
BR033	0.5206	Wild	Wild
BR050	0.5406	Wild	Wild
BR034	0.5696	Wild	Wild
BR045	0.5910	Wild	Wild
BR063	0.6151	Wild	Wild
BR016	0.7157	Wild	Wild
BR024	0.7668	Wild	Wild
BR052	0.7820	Wild	Wild
BR036	0.8019	Wild	Wild
BR027	0.8796	Wild	Wild
BR048	0.9536	Wild	Wild
BR043	1.0681	Wild	Wild
BR058	1.1003	Wild	Wild
BR064	1.2907	Mutant	Mutant
BR040	1.4288	Mutant	Mutant
BR013	1.4350	Mutant	Wild
BR020	1.4504	Mutant	Mutant
BR035	1.4809	Mutant	Mutant
BR026	1.6511	Mutant	Mutant
BR046	1.6968	Mutant	Mutant
BR017	1.7842	Mutant	Mutant
BR010	1.9654	Mutant	Mutant
BR001	2.1603	Mutant	Mutant
BR005	2.1959	Mutant	Mutant
BR021	2.3457	Mutant	Mutant
BR022	2.3744	Mutant	Mutant
BR053	2.4841	Mutant	Mutant
BR011	2.5209	Mutant	Mutant
BR041	3.6260	Mutant	Mutant
BR009	4.0595	Mutant	Mutant

### TP53 signature score for 189 patients in the validation cohort

**Fig. 2:**
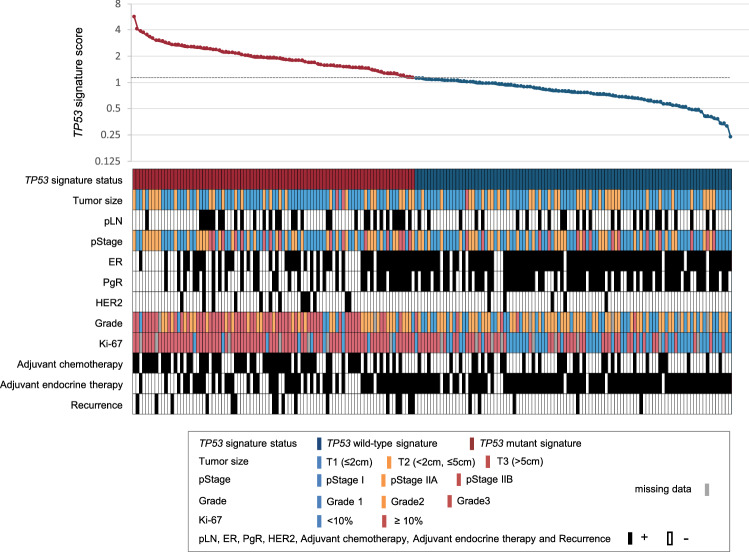
*TP53* signature score and clinicopathological characteristics. The upper graph shows the *TP53* signature score of each case in the validation cohort. Red and blue represents *TP53* mutant signature and *TP53* wild-type signature, respectively. The lower figure shows the clinicopathological characteristics of the corresponding cases in the upper graph. Legend of colors is shown in the figure. pStage, pathological stage; pLN, pathological lymph node; ER, estrogen receptor; PgR, progesterone receptor; HER2, human epidermal growth factor receptor type 2

*TP53* signature scores of 189 cases of the validation cohort were calculated. With use of a cutoff value of 1.11, 89 patients were classified as *TP53* mutant signature, and the remaining 100 cases were classified as *TP53* wild-type signature. Patient characteristics disaggregated by *TP53* status is shown in Table 2 and Fig. 2. A significant difference was observed between the two different *TP53* signatures with respect to ER, PgR, HER2, tumor grade, histological type, Ki-67, postoperative adjuvant chemotherapy, and postoperative adjuvant endocrine therapy.

**Table 2:** Clinicopathological characteristics disaggregated by *TP53* status

	Total		Mutant signature		Wild-type signature		P*
	No. of patients	%	No. of patients	%	No. of patients	%	
Samples	189	100	89	47	100	53	
Age, years (median)	29–98 (58.0)		29–83 (59.0)		26–98 (56.0)		0.077
pStage							0.55
I	95	50	41	46	54	54	
IIA	65	34	33	37	32	32	
IIB	29	15	15	17	14	14	
ER							< 0.0001
+	138	73	49	55	89	89	
−	51	27	40	45	11	11	
PgR							< 0.0001
+	101	54	34	38	67	67	
−	88	46	55	62	33	33	
HER2							0.025
+	18	10	13	15	5	5	
−	171	90	76	85	95	95	
Pathological tumor size, cm							*0.78*
≤ 2	125	66	59	66	66	66	
> 2, ≤ 5	61	32	28	31	33	33	
> 5	3	2	2	2	1	1	
pLN							0.19
+	57	30	31	35	26	26	
−	132	70	58	65	74	74	
Grade							< 0.0001
1	46	25	8	9	38	40	
2	82	45	29	33	53	55	
3	55	30	50	57	5	5	
NA	6		2		4		
Histology							0.043
Invasive ductal carcinoma	179	95	85	96	94	94	
Invasive lobular carcinoma	3	2	0	0	3	3	
Medulary carcinoma	3	2	3	3	0	0	
Mucinous	3	2	0	0	3	3	
Undiff. carcinoma	1	1	1	1	0	0	
Ki-67							< 0.0001
< 10	55	32	9	11	46	51	
≥ 10	118	68	37	89	45	49	
NA	16		7		9		
Adjuvant chemotherapy							< 0.0001
+	86	46	54	61	32	32	
−	103	54	35	39	68	68	
Adjuvant endocrine therapy							< 0.0001
+	137	72	49	55	88	88	
−	52	28	40	45	12	12	

P* Chi-square test was used for statistical analysis of patients' characteristics except for age. Kruskal–Wallis test was used for statistical analysis of patients' age

*pStage* pathological stage, *ER* estrogen receptor, *PgR* progesterone receptor, *HER2* human epidermal growth factor receptor type 2, *pLN* pathological lymph node, *NA* not available

### Recurrence-free survival, overall survival and breast cancer-specific survival by TP53 signature status

**Fig. 3:**
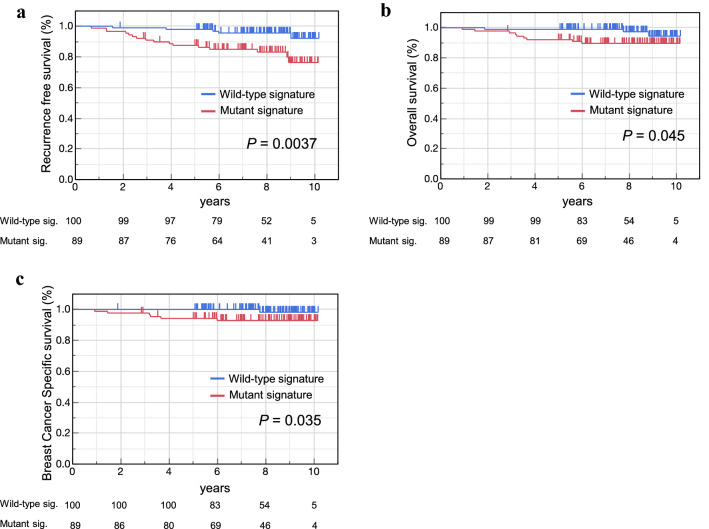
RFS, OS and BCSS by *TP53* signature status. RFS (**a**), OS (**b**), and BCSS (**c**) by *TP53* status based on the *TP53* signature score in the validation cohort were constructed using the Kaplan–Meier method. The differences were compared using the log-rank test. The short vertical line on the curve represent censored. *RFS* recurrence-free survival, *OS* Overall survival, *BCSS* breast cancer-specific survival

The *TP53* mutant signature group showed significantly poorer RFS than that shown by the *TP53* wild-type signature group (Fig. 3a). In OS and BCSS, the *TP53* mutant signature group showed significantly worse than *TP53* wild-type signature group (Fig. 3b, c).

### Univariate and multivariate analyses to identify factors associated with RFS

On univariate analysis, tumor stage, lymph node and *TP53* status by signature were significantly associated with RFS (Table 3). On multivariate analysis, only the *TP53* status by signature showed a significant association with RFS. Our results indicate that *TP53* signature based on multiplex RT–PCR was an independent predictor of RFS.

**Table 3:** Results of uni- and multivariate analysis (Cox proportional hazard model) showing correlation of RFS with clinicopathological factors in patients with breast cancer

Variable	Univariate			Multivariate		
	HR	95% CI	*P*	HR	95% CI	*P*
pStage (vs. Stage I)	2.60	1.01–6.69	0.05	1.72	0.48–6.10	0.40
pLN (vs. negative)	2.55	1.08–6.01	0.03	1.65	0.52–5.17	0.39
Pathological tumor size (vs. T1)	1.27	0.53–3.07	0.59			
Grade (vs. 1–2)	1.14	0.46–2.82	0.78			
ER (vs. positive)	1.33	0.54–3.30	0.54			
PR (vs. positive)	1.54	0.65–3.67	0.32			
HER2 (vs. negative)	0.46	0.06–3.39	0.44			
Ki-67 (vs. < 10%)	4.15	0.95–18.1	0.06			
Adjuvant chemotherapy (vs. non-therapy)	1.47	0.62–3.51	0.38			
Adjuvant endocrinetherapy (vs. non-therapy)	0.97	0.38–2.50	0.95			
*TP53* status by signature (vs. wild-type)	3.96	1.45–10.8	0.01	3.73	1.36–10.20	0.01

*pStage* pathological stage, *pLN* pathological lymph node, *ER* estrogen receptor, *PgR* progesterone receptor, *HER2* human epidermal growth factor receptor type 2, *HR* hazard ratio, *CI* confidence interval

### RFS by TP53 signature status in subtypes of breast cancer

**Fig. 4:**
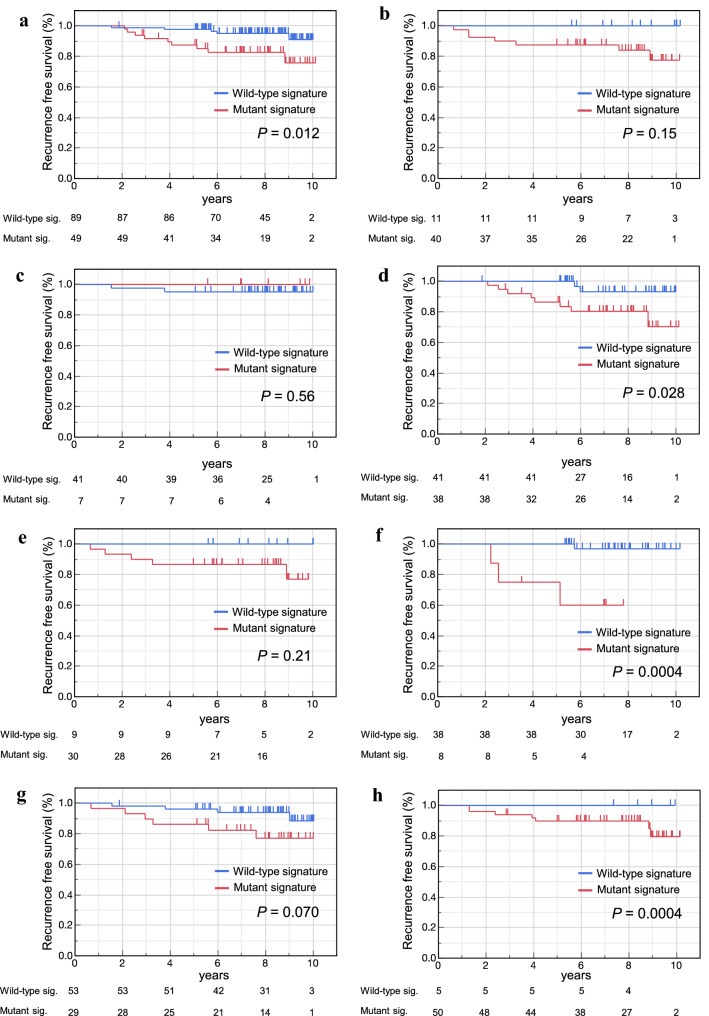
RFS by *TP53* signature status in subtypes of breast cancer and in grade. RFS stratified by *TP53* status based on the *TP53* signature score in patients with ER + (**a**), ER(−) (**b**), Luminal A like (**c**), Luminal B Like (**d**), TNBC (**e**), Grade 1 (**f**), Grade 2 (**g**) and Grade 3 (**h**) were constructed using the Kaplan–Meier method. The differences were compared using the log-rank test. The short vertical line on the curve represent censored. *ER* estrogen receptor, *TNBC* triple negative breast cancer

In ER positive subtype, RFS of *TP53* wild-type signature was significantly better than that of *TP53* mutant signature (*P* = 0.012) (Fig. 4a). Although a significant difference between *TP53* signature status was not shown in ER negative subtype, luminal A like group (ER positive and Ki-67 < 10%) and triple negative breast cancer (TNBC) group, no recurrent cases was observed in *TP53* wild-type signature group (Fig. 4b, c, e). On the other hand, a significant difference was observed between *TP53* signature status in luminal B like subtype (ER positive and Ki-67 ≥ 10%) (Fig. 4d). A survival analysis in HER2 positive subtype did not be carried out because of lack of patients. In grade 1 and 3, RFS of *TP53* mutant signature group was significantly worse than that of wild-type group (Fig. 4f, h). Although the significant difference could not be observed, RFS of *TP53* mutant signature group showed worse trend than that of wild-type group in grade 2 (Fig. 4g).

## Discussion

The *TP53* mutation has long been known as an independent predictor of poor prognosis among patients with breast cancer [10, 11]. To develop a reliable diagnostic kit, we created the gene expression signature that could diagnose the *TP53* gene status using microarray analysis [14]. Uji et al. reported that the *TP53* status determined by gene expression signature was a superior predictor of prognosis than *TP53* status determined on direct DNA sequencing (including the classical Sanger sequencing and the NGS method) [17]. Today, although the *TP53* gene mutation can be analyzed in detail by the cancer genome profiling test, the *TP53* signature is considered to have an advantage in terms of prognosis prediction for breast cancer. Lehmann et al. verified the prognostic predictability of 351 reported gene expression profiles on a meta-analysis based on 31 breast cancer cohorts [18]. They found *TP53* signature was a robust prognostic factor, and was better than well-known gene expression profiles such as OnctypeDX and Mammaprint. Furthermore, Lehmann et al. verified that *TP53* signature was a predictor of therapeutic response in their meta-analysis [18]. Similarly, Oshima et al. reported that signature could predict response to preoperative chemotherapy [19]. As described above, *TP53* signature is confirmed to be both an independent prognostic factor and an independent predictor for response to chemotherapy.

In this report, a simple and easy multiplex RT–PCR diagnostic system for *TP53* signature was developed and the rate of agreement of *TP53* status by *TP53* signature score and the *TP53* status by microarray was enough high (97.1%) (Table 1).

In the validation cohort, a significant difference was observed between the two *TP53* signatures with respect to ER, PgR, HER2, histological grade, Ki-67 histological type, adjuvant chemotherapy and adjuvant endocrine therapy (Table 2). These results do not contradict those reported from previous studies [14, 15, 20, 21].

The *TP53* mutant signature based on the *TP53* signature score was associated with significantly poor RFS, OS and BCSS as compared to that associated with the *TP53* wild-type signature. On univariate and multivariate analysis, *TP53* signature was significantly associated with PFS independent of other clinicopathological factors. These results indicate that the *TP53* status diagnosed by this diagnostic system was an independent prognostic factor in patients with breast cancer for whom curative resection (stage I–II) is performed.

In this report, we showed for the first time that there was clinical significance among breast cancer subtypes and grades. In the ER positive, especially in Luminal B like subgroup, Grade1 and 3 subgroup, it was clearly seen that the prognosis was closely associated with the *TP53* status. In ER negative group, Luminal A like subtype and TNBC, the significant difference was not observed between *TP53* signature status. But, because there was no recurrence in *TP53* wild-type signature group, it can be said that *TP53* signature had clinical significance in these subtypes.

There are some limitations of the interpretation of this study. First, the sample size was relatively small, and the recurrence events were few so far. We are going to follow up recurrent events sequentially. Second, uniform treatment intervention was not carried out for the study cohort because it is an observational, prospective study. We are currently conducting a large scare retrospective-prospective study to confirm the clinical significance of *TP53* signature using several prospective studies conducted in Japan.

In conclusion, we developed a relatively simple multiplex RT–PCR diagnostic system to determine the *TP53* signature. Its diagnostic accuracy and prognostic value were verified in a prospective cohort. And we showed the clinical significance of *TP53* signature among breast cancer subtypes. This simple and precise diagnostic system may help in prognostic assessment, therapeutic decision-making, and treatment optimization in patients with breast cancer.

## Supplementary Information

Below is the link to the electronic supplementary material.

Supplementary file1 (DOCX 38 kb)

## Abbreviations

BCSS: Breast cancer-specific survival
ER: Estrogen receptor
FEC: 5-FU/epirubicin/cyclophosphamide
FF: Fresh frozen
FFPE: Formalin-fixed paraffin embedded
HE: Hematoxylin–Eosin
HER2: Human epidermal growth factor receptor type 2
OS: Overall survival
PCR: Polymerase chain reaction
RFS: Recurrence-free survival
PgR: Progesterone receptor
RT: Reverse transcription
TNBC: Triple negative breast cancer
