# Supplementary material for: TP53 signature diagnostic system using multiplex reverse transcription–polymerase chain reaction system enables prediction of prognosis of breast cancer patients
Source: Breast Cancer. 2021 Jul 24;28(6):1225–34. doi: 10.1007/s12282-021-01250-z (PMC8514380; doi:10.1007/s12282-021-01250-z)
Supplement: Supplementary file 1 [file 12282_2021_1250_MOESM1_ESM.docx]

Supplemental Table 1. Primer set of *TP53* signature

| Gene name | Primer | Primer Sequence | Primer concetration (nM) | Base size of signal peek | Status in mutant [illegible] |
|---|---|---|---|---|---|
| PKMYT1 | forward | AGGTGACACTATAGAATATACAGGGCTCCTATGGGACAG | 500 | 96 | up-regul[illegible] |
| | reverse | GTACGACTCACTATAGGGAACTTCCAGGATGGTGAGGC | 200 | | |
| CENPF | forward | AGGTGACACTATAGAATAAACAGCTGGTGGCAGCAGATC | 7.8 | 100 | up-regul[illegible] |
| | reverse | GTACGACTCACTATAGGGATGCCTGAATCTACTGGGCTCCG | 200 | | |
| ASPM | forward | AGGTGACACTATAGAATACCGTAGACTTGCAATGTGGA | 4 | 103 | up-regul[illegible] |
| | reverse | GTACGACTCACTATAGGGAAGGTCCCAGTTCTGTGTGAGA | 200 | | |
| KIF23 | forward | AGGTGACACTATAGAATAAGAAATAGAAGAGGATCATTGCGGCAGG | 10 | 109 | up-regul[illegible] |
| | reverse | GTACGACTCACTATAGGGACGGAGACGAATTGGTGGTGCGT | 200 | | |
| BIRC5 | forward | AGGTGACACTATAGAATATGGCCCTTCTTGGAGGGCTGCG | 62.5 | 112 | up-regul[illegible] |
| | reverse | GTACGACTCACTATAGGGACTCAGTGGGGCAGTGGATGAAGCC | 200 | | |
| TTC12 | forward | AGGTGACACTATAGAATATGCAAAGGAACGAGCCAAGA | 16 | 118 | down-regu[illegible] |
| | reverse | GTACGACTCACTATAGGGACAGCAAATGCTTCATTCCCT | 200 | | |
| UBE2T | forward | AGGTGACACTATAGAATACAGTGGACAGAGAAGCATGC | 50 | 120 | up-regul[illegible] |
| | reverse | GTACGACTCACTATAGGGAGGAGTCACCAGCCTCTGGTA | 200 | | |
| PTTG1 | forward | AGGTGACACTATAGAATACTAAGGATGGGCTGAAGCTGGGGT | 7.8 | 124 | up-regul[illegible] |
| | reverse | GTACGACTCACTATAGGGATTTTGCCAAAACGTGGTGTTGAAACTTGA | 200 | | |
| RPLP0 | forward | AGGTGACACTATAGAATATCAACGGGTACAAACGAGTC | 2 | 127 | internal c[illegible] |
| | reverse | GTACGACTCACTATAGGGAGATCAGCCAAGAAGGCCTT | 200 | | |
| LIN9 | forward | AGGTGACACTATAGAATAGATAGGACAGGGCTTGGAACCCATACC | 10 | 130 | up-regul[illegible] |
| | reverse | GTACGACTCACTATAGGGAAACACCAAAGGCAGCAATTGGCATTGT | 200 | | |
| CDC45L | forward | AGGTGACACTATAGAATATTCTCTTCGTGGCCTCGGACGTGGAT | 62.5 | 133 | up-regul[illegible] |
| | reverse | GTACGACTCACTATAGGGACTGGAACCAGCGTATATTGCACGTGGTC | 200 | | |
| STATIP1 | forward | AGGTGACACTATAGAATATCCAGAAGGAGCCACTGTCCCTGCATTG | 7.8 | 136 | down-regu[illegible] |
| | reverse | GTACGACTCACTATAGGGAGTTAACAGCTCCTCTTCATCAGAAGGCTGAG | 200 | | |
| C10orf3 | forward | AGGTGACACTATAGAATAAGTTGTCTGCTGCAACCTCACGAATTGCT | 7.8 | 139 | up-regul[illegible] |
| | reverse | GTACGACTCACTATAGGGATTATTGATGAGTTGAAGCAGTTTGGAGCCAC | 200 | | |
| PSMC4 | forward | AGGTGACACTATAGAATACTTGGAAGACTATGTGGCCCG | 2.5 | 143 | internal c[illegible] |
| | reverse | GTACGACTCACTATAGGGACAATGTAGCGGTTTTCACGG | 200 | | |
| FLJ10719 | forward | AGGTGACACTATAGAATACAGTTTGTCCAAGTTACTGGAGCCCTCCTCTCC | 25 | 145 | up-regul[illegible] |
| | reverse | GTACGACTCACTATAGGGATTGCAAAACAAGGCATCCTCCCGGCTGT | 200 | | |
| MGC7036 | forward | AGGTGACACTATAGAATACAGGAGCTAAGGGATGTGCT | 1.5 | 148 | down-regu[illegible] |
| | reverse | GTACGACTCACTATAGGGAGCCTTCTCTTGGTGGAATCA | 200 | | |
| HEXIM1 | forward | AGGTGACACTATAGAATAGGGATTTAACCCTTTGTGGATCT | 15.6 | 151 | down-regu[illegible] |
| | reverse | GTACGACTCACTATAGGGAGTTGGTGAGCAAGGTGATG | 200 | | |
| MKNK2 | forward | AGGTGACACTATAGAATAGTGCTGGGGGAGGGCGCTCATGCCCGAGT | 50 | 154 | down-regu[illegible] |
| | reverse | GTACGACTCACTATAGGGAGAAAACCCTGCTCCGAATGTGGCCTGGCT | 200 | | |
| CDCA8 | forward | AGGTGACACTATAGAATAGCACAGCGAGGTTTTGCTCAGCCCTTGTCT | 1 | 157 | up-regul[illegible] |
| | reverse | GTACGACTCACTATAGGGACGAGCTTCCGCCTCCGTAAGGAGTTGGTC | 200 | | |
| KNTC2 | forward | AGGTGACACTATAGAATATCCTTGGAGAAACACAAGCA | 500 | 160 | up-regul[illegible] |
| | reverse | GTACGACTCACTATAGGGATTCTTCAGTCGTGGTTTGCA | 200 | | |
| GMNN | forward | AGGTGACACTATAGAATAAATAAAAATCTTGGAGGAGTCACCCAGGAGTCA | 12.5 | 163 | up-regul[illegible] |
| | reverse | GTACGACTCACTATAGGGATTCCTTAAGTGCTTCATACAGCGCCTTTCTCCGT | 200 | | |
| TMEM25 | forward | AGGTGACACTATAGAATAAGCACCTTCACTGTCACTGC | 100 | 168 | down-regu[illegible] |
| | reverse | GTACGACTCACTATAGGGAACTTGGGCAATCTCTGGCTT | 200 | | |
| TBP | forward | AGGTGACACTATAGAATAAGTGAGACGAGTTCCAGCGCA | 125 | 170 | internal c[illegible] |
| | reverse | GTACGACTCACTATAGGGAGGGATTCCGGGAGTCATGGCA | 200 | | |
| PTP4A2 | forward | AGGTGACACTATAGAATAACTTAAGAAGTATGGAGTGACGACTT | 1.5 | 173 | down-regu[illegible] |
| | reverse | GTACGACTCACTATAGGGACCAAATAAAGCAGCTGTTTGGAATTG | 200 | | |
| HCAP-G | forward | AGGTGACACTATAGAATAGCACCAGAACCAGGCGAAG | 125 | 180 | up-regul[illegible] |
| | reverse | GTACGACTCACTATAGGGACCCTCTCCACAGCTGGTTCACGTTTATAGACCACCA | 200 | | |
| PLK1 | forward | AGGTGACACTATAGAATATAGAGACGCGGGCTCAGGCA | 62.5 | [illegible] | [illegible] |
